# Enhanced interaction between natural killer cells and lung cancer cells: involvement in gefitinib-mediated immunoregulation

**DOI:** 10.1186/1479-5876-11-186

**Published:** 2013-08-12

**Authors:** Sisi He, Tao Yin, Dan Li, Xiang Gao, Yang Wan, Xuelei Ma, Tinghong Ye, Fuchun Guo, Jianhong Sun, Ziqiang Lin, Yongsheng Wang

**Affiliations:** 1 Department of Thoracic Oncology, Cancer Center, State Key Laboratory of Biotherapy, West China Hospital, Sichuan University, Chengdu 610041, PR China

**Keywords:** Gefitinib, Natural killer cells, Immunotherapy, EGFR, NSCLC

## Abstract

**Background:**

Natural killer (NK) cells can kill tumor cells in a non-MHC-restricted manner. However, cancer cells frequently escape from the attack of NK cells by multiple ways. In this study, we investigated the effect of gefitinib on the interaction between NK cells and lung cancer cells.

**Methods:**

^51^Cr release assay, CD107a assay, and IFN-γ secretion assay were performed to detect the sensitivity of lung cancer cell lines A549 and H1975 to NK cells cytotoxicity in the presence of gefitinib. Human NK cells were co-cultured with A549 and H1975 cell lines in the presence of gefitinib. NKG2D ligands, ULBP1, ULBP2, MICA, and MHC-I on tumor cells, and NKG2D, NKp44 and NKp46 on NK cells were evaluated with flow cytometry. ^51^Cr release assay was performed when NKG2D antibody were added into the co-culture system. Expressions of stat3 and LC3 I/II on tumor cells were determined with western blot after co-cultured with NK cells. After treated with gefitinib, mannose-6-phosphate receptor (MPR) on H1975 cells was evaluated by flow cytometry. ^51^Cr release assay were performed when MPR antagonist were used.

**Results:**

Gefitinib increased cytotoxicity of NK cells to human lung cancer H1975 cells with EGFR L858R + T790M mutations, while not in A549 cells with wild type EGFR. Gefitinib could block the immune escape by up-regulating the expression of NKG2D ligands ULBP1, ULBP2 or MICA on tumor cells and NKG2D on NK cells in the co-culture system. Gefitinib and NK cells up-regulated MHC-I expression in A549 while not in H1975 cells. NKG2D antibody blocked the enhanced NK cytotoxicity by gefitinib. The combination of NK cells and gefitinib could significantly down-regulate stat3 expression. Furthermore, NK cells-mediated tumor cell autophagy was observed in A549 cells while not in H1975 cells. Notably, gefitinib increased autophagy and MPR expression in H1975 cells, which improved the sensitivity to NK cell-based immunotherapy.

**Conclusions:**

Gefitinib greatly enhanced NK cell cytotoxicity to lung cancer cells with EGFR L858R + T790M resistance mutation. Combination of EGFR tyrokinase inhibitors and NK cells adoptive immunotherapy may represent a potentially effective strategy for patients with non-small cell lung cancer.

## Background

Lung cancer is a leading cancer death worldwide [1]. The use of selectively targeted agents has revolutionized the treatment of lung cancer and shown promising clinical activity. EGFR is frequently over-expressed in non-small cell lung cancers (NSCLC) [2]. As the first small inhibitor for EGFR, gefitinib induce dramatic clinical responses and improve progression-free survival, through inhibition of EGFR-driven signals for tumor cells survival and proliferation [3]. However, many cancer patients invariably develop drug resistance [4-6]. The secondary T790M mutation within the EGFR kinase domain is a major mechanism of acquired resistance to EGFR tyrosine kinase inhibitors (TKI) in NSCLC [7]. However, clinical response to gefitinib has been demonstrated to be not correlated with EGFR levels, and several other molecular mechanisms are also important in predicting clinical response [8,9].

NK cells are key components of innate immunity and participate in immunity against virus-infected and neoplastic cells [10]. NK cell-based immunotherapy may be an efficient way to eliminate tumor cells, and many clinical trials have been conducted and showed benefit [11]. NK cell can kill many cancer cells via direct killing, induction of apoptosis or IFN-γ secretion [12,13]. Furthermore, NK cells can inhibit tumor cell metastasis [14]. Several activating receptors on NK cell surface have been discovered, which are dispensable for NK cell activation [15,16]. The major receptors responsible for NK cells activation are NKG2D and natural cytotoxicity receptors (NCRs; that is, NKp30, NKp44 and NKp46) [17]. NKG2D is the main activating receptor, and the binding to its ligand can promote NK cells cytotoxic lysis of target cells. Engagement of NKG2D activates NK cells and then become a promising anti-cancer strategy [18,19]. MHC class I chain-related molecules, MICA and MICB, and the UL16-binding proteins, ULBP-1, ULBP-2, and ULBP-3 are the main ligands for human NKG2D, which expressed on many cancer cells and infected cells [20,21]. Several clinical interventions have been demonstrated to up-regulate NKG2D ligands expression on tumor cells and enhance susceptibility to NK cells, including chemotherapy, radiotherapy and HDAC-1 [22], Proteasome inhibitor [23].

However, several factors limited the efficiency of NK cells adoptive therapy. Except for its poor ability to home to tumor area, tumor microenvironment edited NK cells and changed NK cell response [24-26]. Recent reports showed that melanoma cells inhibited the expression of NK receptors and impaired NK cells cytolytic functions [27]. NK cells per se can induce target cell autophagy and enhance cancer cell survival [28]. Those results suggested that immunosuppressive barriers developed by tumor cells could impair NK cells based immunotherapy.

Several immunomodulatory approaches have been investigated to enhance anti-tumor therapy efficiency. Imatinib potentiates antitumor T cell responses through the inhibition of IDO [29]. Imatinib can act on host DCs to promote NK cell activation [30]. In the present study, we examine how gefitinib modulate the tumor cells and NK cells after short-term interactions. We here show that gefitinib enhance NK cells and tumor cells interaction by modulation of NKG2D ligands and NKG2D and improve anti-tumor NK response. Gefitinib can reduce stat3 expression in tumor cells. MPR expression-induced by gefitinib can facilitate NK cell cytotoxicity in human lung cancer cells with EGFR L858R + T790M resistance mutation. Our results suggest that making use of immunoregulatory property of gefitinib may be a potential new therapeutical option for lung cancer with EGFR L858 + T790M resistance mutation.

## Materials and methods

### Cell culture

Human NSCLC cell lines A549 and H1975 were obtained from American Type Culture Collection (ATCC; Manassas, VA) and maintained in RPMI 1640 media (Hyclone) supplemented with 10% FBS (GIBCO). NK cells were obtained from peripheral blood of different health donors by magnetic bead isolation using NK isolation kit (R&D systems) according to the manufacturer’s instructions. NK cell purity was >85%. All of the researches were performed in accordance with the Sichuan University’s Ethics Committees. NK cells were maintained in RPMI 1640 media supplemented with 200 U/ml IL-2 and 10 ng/ml IL-15 and 10% FBS.

### Flow cytometry

Primary NK cells were stained with CD56 (R&D systems) and CD3 antibodies (BD Pharmingen). Cells were acquired on a FACSCalibur flow cytometer and data were analyzed using Cell Quest software (BD Biosciences). NK cells were co-cultured with the indicated target cells in a ratio of 1:1 in 24-well plates for 24 hours, and 5 μg/ml gefitinib (Astra Zeneca) was added into co-culture system for another 24 hours. Afterward, NK cells were collected and examined for the expression of NKG2D, NKp44, and NKp46. ULBP1, ULBP2, MICA expression were evaluated on tumor cells. Intracellular IFN-γ staining was performed after fixation in 2% paraformaldehyde and permeabilization in 1% Trixton. IFN-γ-PE antibody was purchased from BD Pharmingen. Antibodies against NKG2D, NKp44, NKp46, ULBP1, ULBP2, MICA were purchased from R&D systems. For MPR expression, H1975 tumor cells were treated with gefitinib for 48 hours, and then the MPR levels on cell surface was evaluated by flowcytometry.

### CD107a assays

NK cells were co-cultured with the indicated target cells in a ratio of 1:1 in the presence CD107a antibody (R&D systems) for 4 h in the presence or absence of 5 μg/ml gefitinib. Afterward, cells were washed and CD107a levels on the NK cells were then analyzed by flow cytometry.

### Western blot

Tumor cells were harvested and lysed in radio-immunoprecipitation buffer for 30 min. Protein concentration was determined by Bradford assay. Cell lysates were resolved by SDS-PAGE, and transferred to PVDF membrane (Millipore). Membrane was blocked in 5% non-fat milk and then blots were probed with antibodies for stat3 and LC3 (Cell Signaling Technology) respectively. After incubated with horseradish peroxidase-conjugated secondary antibodies, probes were visualized by a chemiluminescent detection system. GAPDH as a loading control. Antibody against GAPDH was obtained from Cell Signaling Technology.

### ^51^Cr release assay

Target cells were labeled with 1 mCi of Na_2_ ^51^CrO_4_ for 1 h at 37°C. Cells were then washed three times with complete medium and incubated with effector cells at different E:T ratios in the presence or absence of 5 μg/ml gefitinib. After incubation for 4 h at 37°C, cell free supernatants were collected and counted on scintillation counter. Percentage of cytolysis was calculated by (sample release–spontaneous release)/(maximum release–spontaneous release). To block the cytotoxicity of NK cells, mannose-6-phosphate or 20 μg/mL NKG2D antibody (R&D system) were added into the ^51^Cr release assay system.

### Statistical analyses

ANOVA was used to identify significant group differences. *p* < 0.05 was considered statistically significant.

## Results

### Gefitinib enhanced cytotoxicity of NK cells in human lung cancer cells with EGFR L858R + T790M mutation

**Figure 1:**
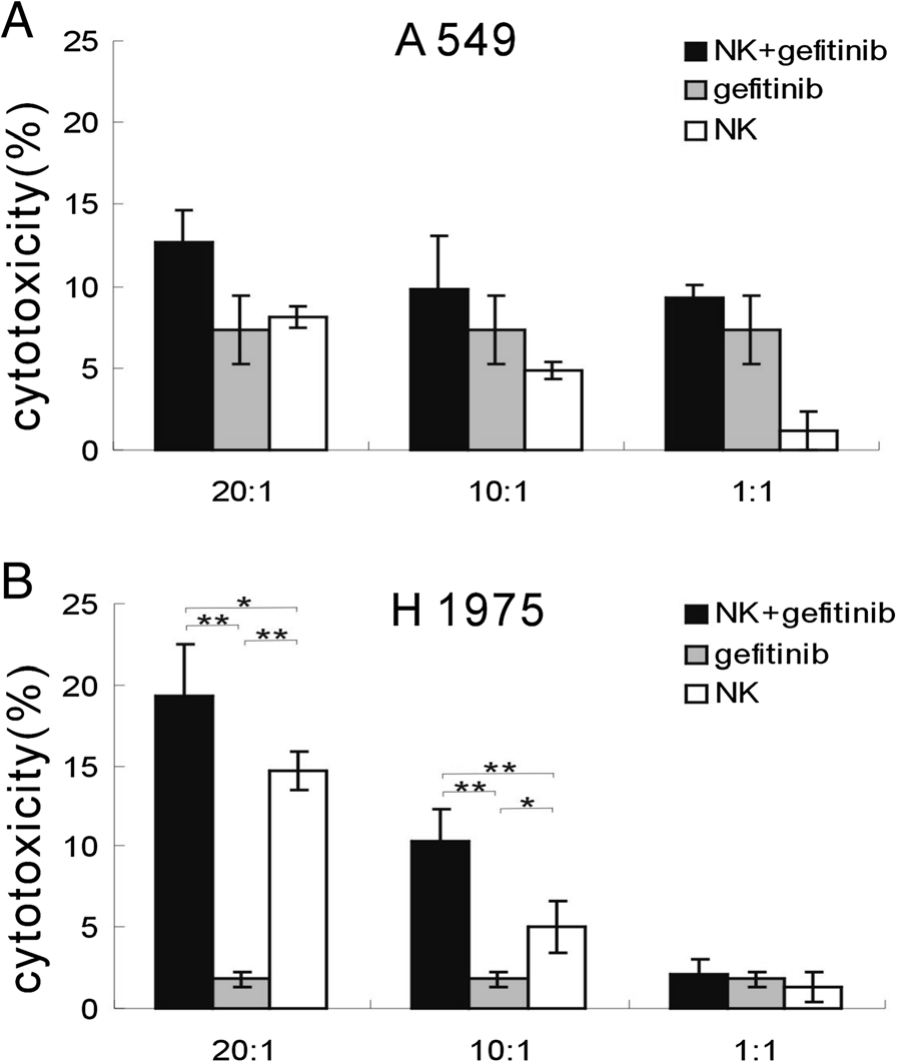
**Gefitinib enhances cytotoxic activity of NK cells to human lung cancer cells. (A-B)**, Primary human NK cells with high purity were co-cultured with A549 **(A)** or H1975 tumor cells **(B)** in the absence and presence of gefitinib for 4 hours. Cytotoxicity was assessed using the ^51^Cr release assay. Data are expressed as means ± SEM. * *p* < 0.05 , **p < 0.01.

To investigate whether gefitinib could increase the susceptibility of NSCLC cell lines to cytolytic activity of NK cells, ^51^Cr releasing assay was performed. Two gefitinib resistance NSCLC cell lines A549 (EGFR wt) and H1975 (EGFR L858R + T790M) were used. In the presence of gefitinib, A549 showed some more enhanced susceptibility to NK cells cytotxicity; however, there were no significant difference (Figure 1A). As to H1975 with L858R + T790M, gefitinib significantly improved NK cells cytotxicity (Figure 1B). Those results suggested that gefitinib enhanced cytotoxicity of NK cells to human lung cancer with EGFR L858R + T790M.

### Degranulation of NK cells triggered by gefitinib

**Figure 2:**
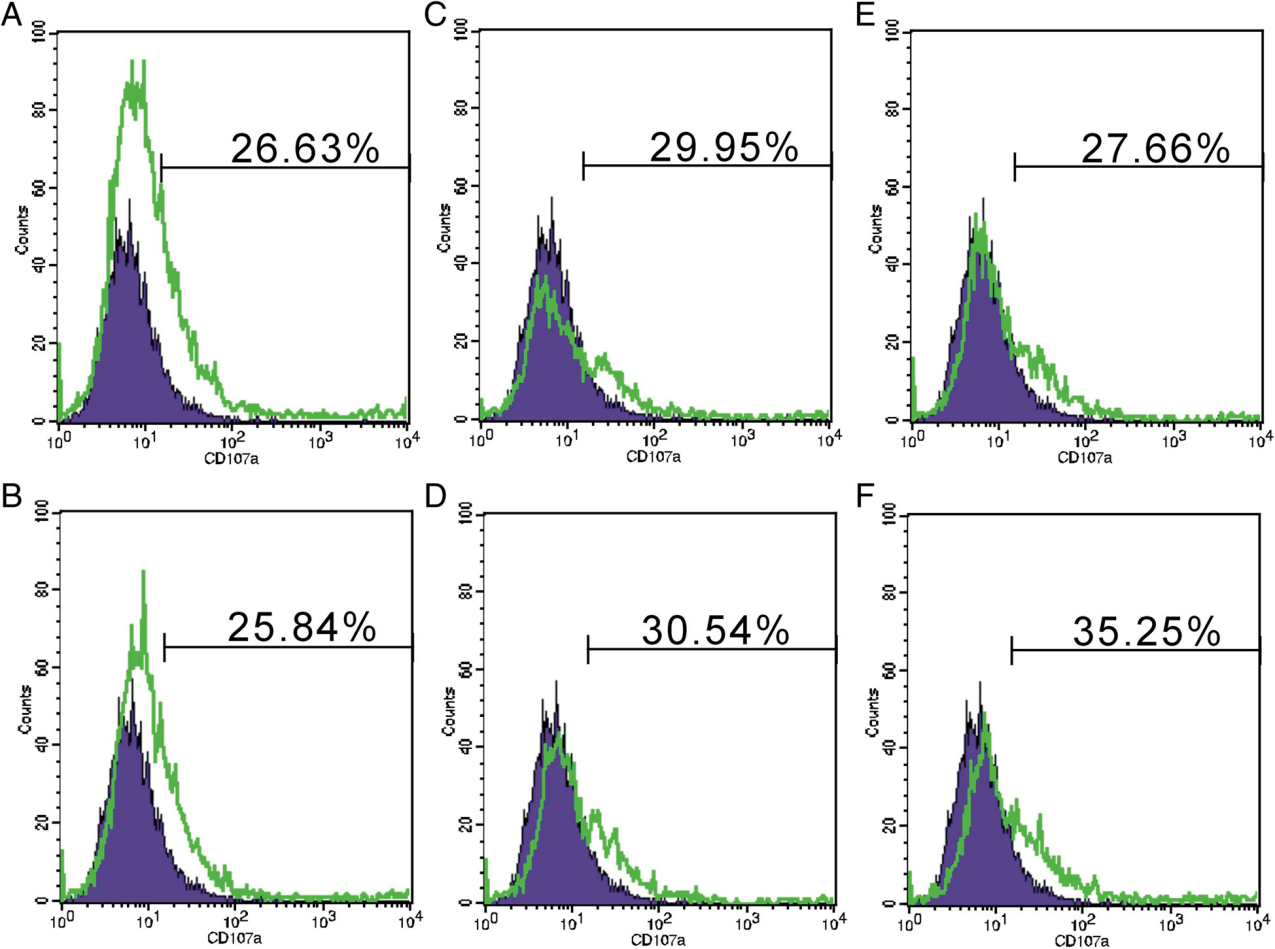
**Gefitinib enhances NK cells degranulation. (A-B)**, NK cells was evaluated for CD107a levels in the absence **(A)** or presence **(B)** of gefitinib. **(C-D)**, NK cells were incubated for 4 h with A549 cell lines together with a CD107a antibody in the absence **(C)** or presence **(D)** of gefitinib. **(E-F)**, NK cells were incubated for 4 h with H1975 cell lines together with a CD107a antibody in the absence **(E)** or presence **(F)** of gefitinib. CD107a levels were evaluated with flow cytometry. Two to three independent experiments were performed.

CD107a degranulation was correlated with NK and T cell killing [31]. The function of NK cells was evaluated by measuring degranulation on the basis of CD107a staining. In the presence of gefitinib, NK cells co-incubated with H1975 degranulated more than did NK cells from control group (Figure 2E-F). However, there was no significant improvement in A549 cells (Figure 2C-D). Our results suggested that gefitinib could enhance the ability of NK cell degradulation to lung cancer cells with EGFR L858R + T790M.

### Role of IFN-γ in the immunomodulation of gefitinib

**Figure 3:**
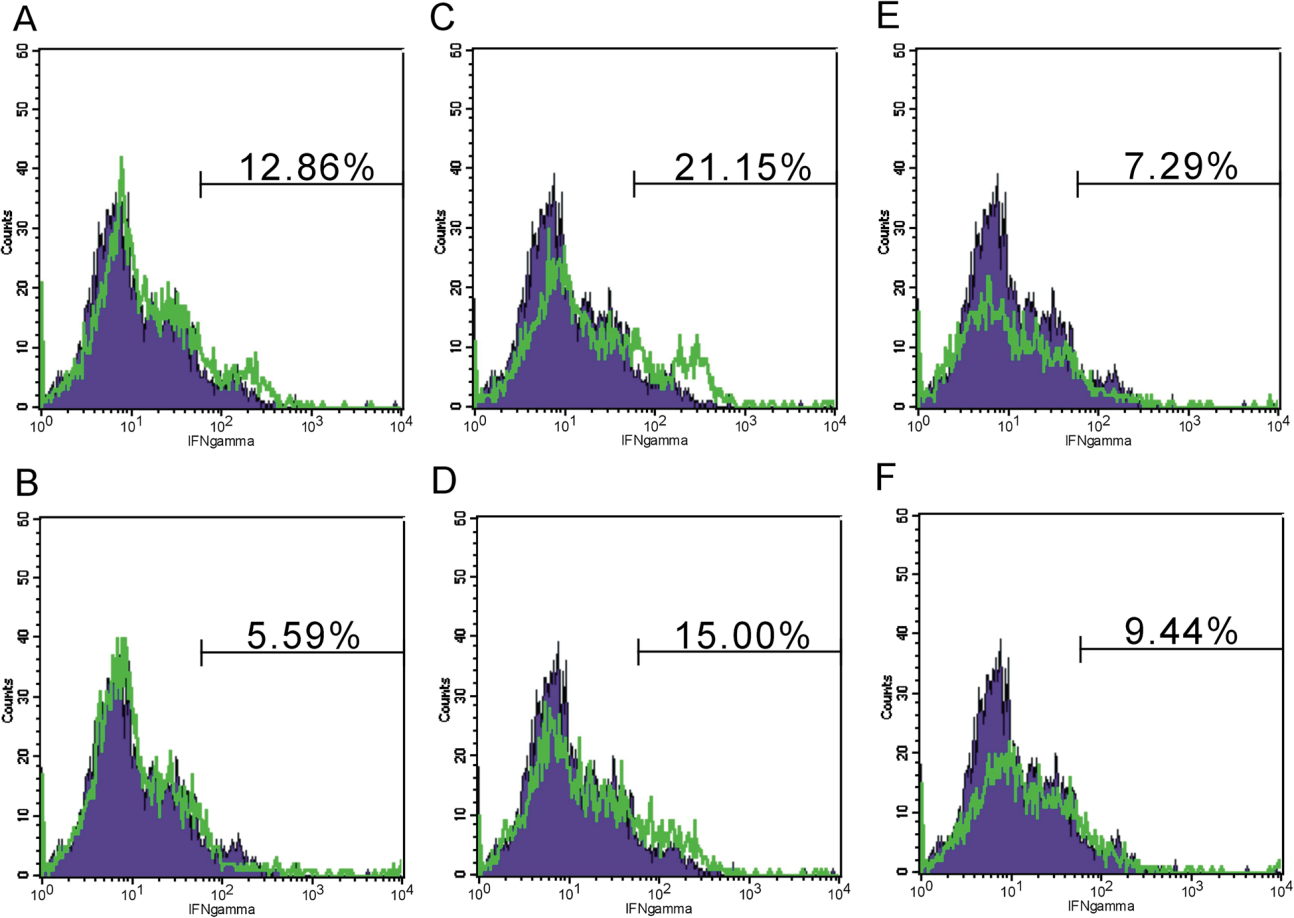
**Gefitinib increases IFN-γ production of NK cells. (A-B)**, NK cells was evaluated for the IFN-γ secretion in the absence **(A)** or presence **(B)** of gefitinib. **(C-D)**, NK cells were incubated with A549 cell lines in the absence **(C)** or presence **(D)** of gefitinib. **(E-F)**, NK cells were incubated with H1975 cell lines in the absence **(E)** or presence **(F)** of gefitinib. Intracellular IFN-γ levels were evaluated with flow cytometry. Two independent experiments were performed.

IFN-γ has been demonstrated to be an important effector cytokine produced by NK cells, which plays an essential role in response to infection and tumors [32]. To determine whether gefitinib enhancement of NK cell cytotoxicity was partially attributed to IFN-γ, we then evaluated IFN-γ expression by NK cells. There were no any improvements in IFN-γ secretion in A549 cells (Figure 3C-D). H1975 tumor cells inhibited IFN-γ secretion from the NK cells (Figure 3E). However, gefitinib significantly attenuated the inhibitory effect of H1975 cells on NK cells IFN-γ secretion by after 24 hours stimulation (Figure 3F).

### Gefitinib restore receptor-ligand interactions between NK cells and human lung cancer cells

**Figure 4:**
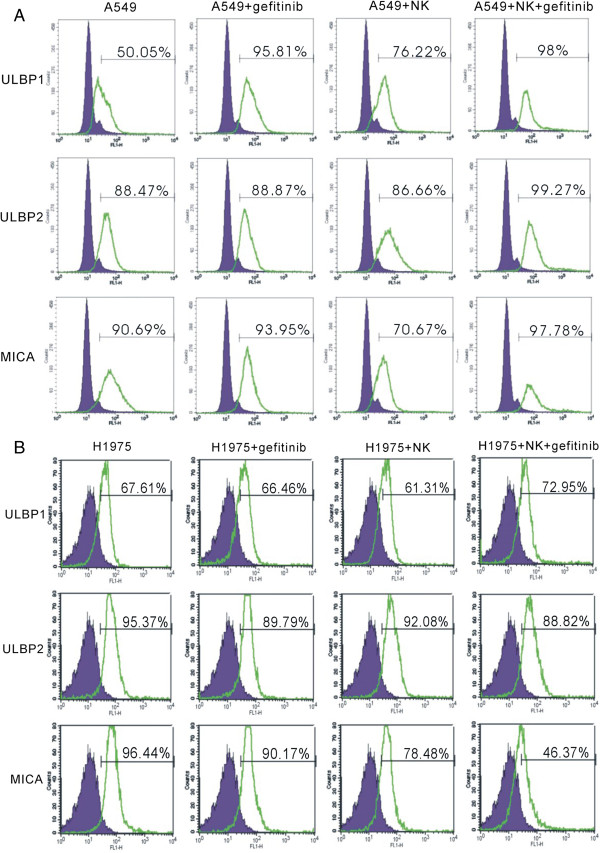
**Gefitinib up-regulates NKG2Dligands on tumor cells. (A-B)**, ULBP1, ULBP2 and MICA expression was evaluated on A549 **(A)** and H1975 **(B)** tumor cells after co-culture with NK cells in the presence or absence of gefitinib. Two independent experiments were performed.

Tumor cells impair NK cell-mediated killing by decreasing expression of surface ligands for NK cell activating receptors, which include NKG2D and NCRs [33,34]. To investigate whether gefitinib could up-regulate surface ligands for NK cell activating receptors, we co-cultured two human lung cancer cell lines with NK cells and evaluated the expression of ULBP1, ULBP2 and MICA by flowcytometry. ULBP1, ULBP2 and MICA were down-regulated after co-culture of NK cells and H1975 cell line (Figure 4B). In A549, ULBP2 and MICA expression were down-regulated (Figure 4A). Those results suggested that human lung cancer cells could decrease expression of surface ligands for NKG2D. However, once gefitinib was administered, ULBP1, ULBP2 and MICA were all up-regulated in A549 cells (Figure 4A). In the H1975 cell line, gefitinib could only up-regulate ULBP1 expression (Figure 4B). Our resultes suggested that gefitinib could partially increase expression of surface ligands for NKG2D and enhance immune recognition of cancer cells by NK cells.

**Figure 5:**
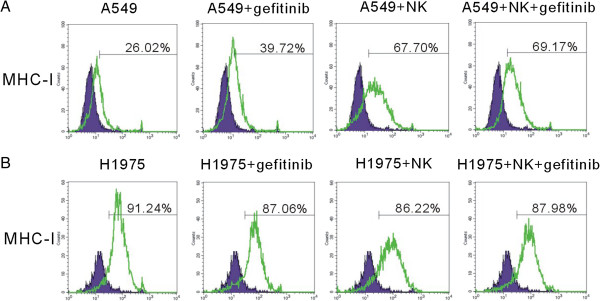
**Gefitinib changes MHC-I expression on tumor cells. (A-B)**, MHC-I expression was evaluated on A549 **(A)** and H1975 **(B)** tumor cells after co-culture with NK cells in the presence or absence of gefitinib. Two independent experiments were performed.

To investigate whether gefitinib influence the MHC-I expression during the short interaction between NK cells and tumor cells, we evaluated the MHC-I levels on tumor cells. In A549 cell line, gefitinib and NK strikingly up-regulated the MHC-I expression (Figure 5), while the expression of MHC-I was slightly down-regulated in H1975 cell line (Figure 5). Collectively, these results suggested that gefitinib and NK cells could up-regulate the MHC-I in human lung cells with wild type EGFR, while not significantly influence the MHC-I expression on human lung cells with wild type EGFR L858R + T790M.

**Figure 6:**
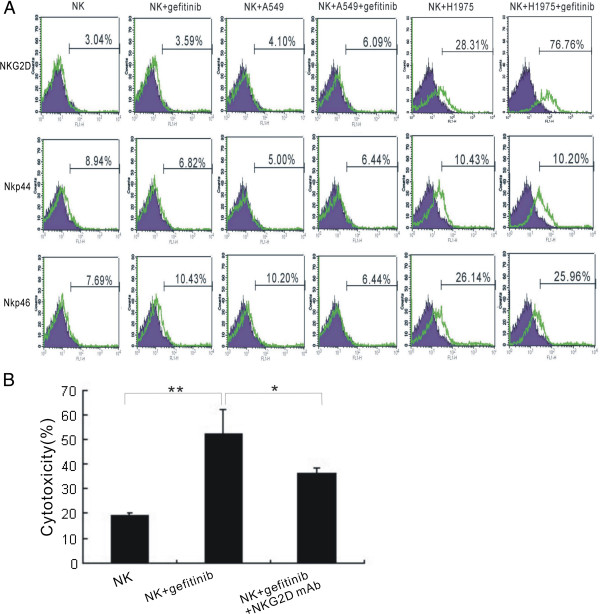
**Gefitinib enhances NK cells cytotoxicity via NKG2D. (A)** Gefitinib up-regulated NKG2D on NK cells. NKG2D, NKp44 and NKp46 expression was examined on NK cells following co-culture with A549 and H1975 tumor cells. Three independent experiments were performed. **(B)** NKG2D antibody blocked the enhanced NK cells cytotoxicity by gefitinib. The concentration of NKG2D antibody was 20 μg/mL. * *p* < 0.05, **p < 0.01.

On the other side, to investigate whether gefitinib could affect NCRs and NKG2D expression on NK cells, we detected NCRs and NKG2D expression by flow cytometry. NCRs had no significant changes; however, we found that in the presence of gefitinib, NKG2D was significantly up-regulated, especially after co-cultured with H1975 tumor cells (Figure 6A). To evaluate whether NKG2D mediated the enhanced cytotoxicity of NK cells by gefitinib, NKG2D antibody was added into the co-culture system. ^51^Cr release assay showed that NKG2D antibody significantly blocked the enhanced cytotoxicity of NK cells by gefitinib (Figure 6B).

### Role of stat3 in the immunomodulation of gefitinib

**Figure 7:**
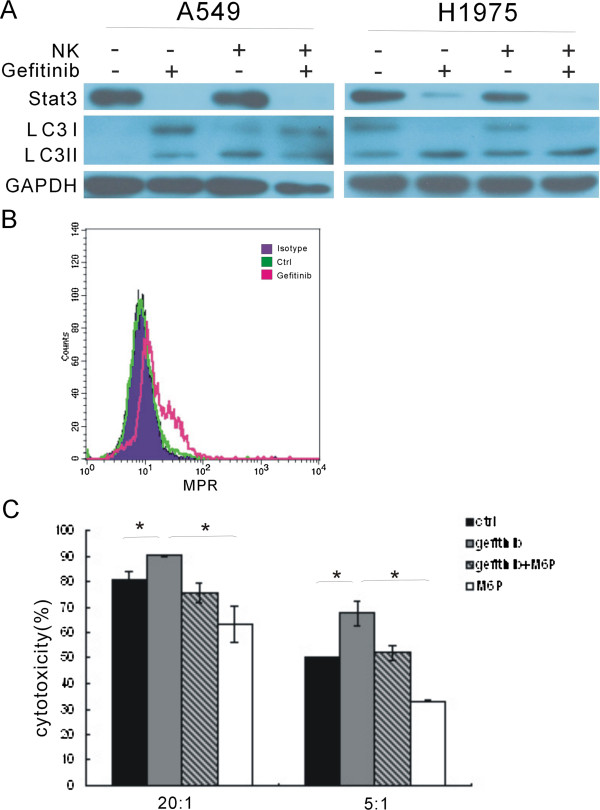
**Stat3, autophagy and MPR expression are engaged in the immunoregulation of gefitinib. (A)**, after co-culture with NK cells in the presence or absence of gefitinib, A549 and H1975 cells were evaluated for stat3 expression and LC3 levels with western blot. **(B)**, MPR expression was elevated on H1975 tumor cells treated with gefitinib. **(C)**, MPR antagonist mannose-6-phosphate impaired the cytotoxic function of human NK cells to H1975 tumor cells treated with gefitinib. Cytotoxicity was assessed with the ^51^Cr release assay. Data are expressed as means ± SEM. * *p* < 0.05.

Activation of Stat3 has been demonstrated in a variety of tumors. Stat3 can be phosphorylated by activated EGFR and promote tumor survival in vivo in NSCLC [35]. Stat3 is a key factor in gefitinib-resistant EGFR T790M cells [36]. Recent reports have demonstrated that Stat3 exerts an inhibitory effect on antitumor NK cell immunity [37]. To determine if gefitinib reversal of tumor cells mediated inhibition of NK cell activation was associated with the inhibition of stat3, we quantified the expression of stat3 in the tumor cells with western blot. As expected, gefitinib treatment alone for 24 hours substantially decreases stat3 expression (Figure 7A). Combination of gefitinib with NK cells can further down-regulate stat3 in H1975 cells (Figure 7A).

### MPR expression-induced by gefitinib enhanced the NK cytotoxity

Although gefitinib could restore NKG2D receptor-ligand interactions between NK cells and human lung cancer cells, and inhibit stat3 expression, further molecular mechanisms should be investigated on the difference between A549 and H1975 to the sensitivity to gefitinib-mediated NK cells response. Recent report suggested that autophagy induced by conventional chemotherapy could mediate tumor cell sensitivity to immunotherapy [38]. To test whether the response difference was caused by autophagy, autophagic marker LC3 was evaluated. We found that gefitinib could increase autophagy in H1975, as demonstrated by the enhanced conversion of LC3-I to LC3-II (Figure 7A), While there was no obvious autophagy in A549 (Figure 7A). Interestingly, we also found that NK cells per se induced autophagy in A549 cells, while not in H1975 cells (Figure 7A).

Autophagy can induce mannose-6-phosphate receptor expression in murine tumor cells [38]. To test whether gefitinib-induced autophagy can up-regulate MPR expression on human tumor cells, we treated H1975 cells for 48 hours with gefitinib and the analyzed the cell membrane MPR expression by flow cytometry. We found that MPR expression was obviously up-regulated after gefitinib treatment (Figure 7B). Then, we further investigated whether gefitinib-induced MPR expression could enhance the cytotoxicity of NK cells. We used MPR antagonist mannose-6-phosphate to block MPR and performed the ^51^Cr releasing assay. MPR blockade significantly impaired the cytotoxic function of NK cells (Figure 7C). Together, these results suggested that MPR expression-induced by gefitinib could enhance the NK cytotoxity.

## Discussion

Reasons for the failure of immune cell based therapy have been advanced [39]. Tumor cells can employ a variety of mechanisms to evade immune surveillance. In our short-term co-culture system, A549 and H1975 lung cancer cells down-regulated surface expression of NKG2D ligands ULBP1, ULBP2 and MICA following co-culture with NK cells. Those ligands facilitate NK cells recognition of tumor cells and render tumor cells susceptible to NK cell-mediated cytolysis [40]. Down regulation of those ligands may help to evade NKG2D-mediated immunosurveillance. NKG2D ligands may represent a potential target for evoking the innate immune response against tumors. Approaches to activate NK cells by up-regulating of NKG2D ligands on tumor cells have been investigated. Our present study and those of others showed that geftinib can partially up-regulate NKG2D ligands ULBP1, ULBP2 or MICA on tumor cells [34]. We also found gefitinib or NK cells could enhance MHC-I expression, which impairs the recognization of NK cells, in lung tumor cells with wild type EGFR, while not in those with EGFR L858R + T790M. NKG2D is the main activation receptor that potently stimulates cytotoxicity and production of IFN-γ by NK cells [41].

Lymphocyte activation integrates multiple signals. NK cells express a plethora of cell surface markers belonging to the TNFR family, such as CD27, CD137 (4-1BB), CD134 (OX40) and glucocorticoid-induced TNFR (GITR), which play important roles in immune synapses [42]. CD137-specific agonist antibodys increase trastuzumab-mediated NK cell cytotoxicity and enhance trastuzumab efficacy against human breast cancer [43]. The other known activating NK cell receptors include NKG2D, NCRs, 2B4, NTB-A and NKp80, CS1 [44] and the leukocyte adhesion molecule DNAM-1 [45]. Here, we focus our study on NKG2D and NCRs, which are recognized as the main triggering receptors of NK cells that are involved in target cell lysis. NCRs recognizes yet uncharacterized ligands on tumor cells. We here found the gefitinib up-regulated markedly NKG2D levels on human NK cells in the co-culture of human H1975 lung cancer cells, while NKp44 and NKp46 expression was less influenced. NKG2D plays an important role in immunosurveillance. Aberrant loss of NKG2D in cancer is a important mechanism of immune evasion. Reduced expression of NKG2D on NK and T cells of cancer patients has been reported [40,46]. We then examined NKG2D expression on NK cells and found that geftinib up-regulated NKG2D expression on NK cells, and we further found that the enhanced NK cytotoxicity by gefitinib was mediated by NKG2D. The functional relevance of restoration of NKG2D–NKG2DL interaction by gefitinib was demonstrated by the enhanced cytotoxicity, degranulation and IFN-γ production of NK cells in response to lung cancer cells with EGFR L858R + T790M resistance mutation.

Recently, immune system has been demonstrated to contribute substantially to the antitumor effects of small molecule inhibitors. Through the inhibition of IDO, imatinib potentiates antitumor T cell responses in gastrointestinal stromal tumor [29]. Imatinib can also act on host DCs to promote NK cell activation [30]. In our present work, we find that, beyond its EGFR tyrokinase inhibitory effect, gefitinib also has immunomodulatory effect in gefitinib-resistance cell lines, which can enhance immune recognization of tumor cells by NK cells and attenuate the inhibitory effect of tumor cells on NK cells.

One of the major reasons for the weak effect of cell based immunotherapy is thought to be immunosuppression. Tumor microenvironment, with abundant of immunosuppressive cells and molecules, can inhibit effector cells and result in insufficient antitumor effects [24-26]. Stat3 plays an important role in the process in tumor immunosuppression. Activation of IL-6R/JAK1/STAT3 signaling can induce de novo resistance in NSCLC with T790M resistance mutation [47]. Activation of stat3 has been demonstrated to lead to the production of multiple immunosuppressive cytokines [48]. Stat3 exerts an inhibitory effect on antitumor NK cell immunity, and Stat3 knockdown decreases MHC class I expression on lung tumor cells and results in the activation of NK cell-mediated cytotoxicity [37]. We found that gefitinib could inhibit stat3 expression in lung cancer cells. Furthermore, combination of gefitinib and NK cells can further reduce stat3 expression. We postulate that the attenuation of inhibitory effect of tumor cells on NK cells may partially attributed to the stat3 inhibition by gefitinib.

In our present study, we also find that high purity NK cells increase autophagy in A549 cancer cells with wide type EGFR, while not in H1975 cells with EGFR L858R + T790M. Lymphocyte provides lytic signals to tumor cells, and they also promote autophagy in the remaining tumor cells. These processes are primarily mediated by NK cells [28]. Cell-mediated autophagy promotes cancer cell survival and induces resistance to subsequent therapies [28]. NK cell-induced autophagic change may promote cancer cells survival. From the perspective of view, NK cells therapy alone may not be an effective strategy. Though gefitinib can also restore NKG2D ligands and NKG2D interaction, and inhibit stat3 expression, we did not find significant improvement on NK cells cytotoxicity to A549 cells with wild type EGFR, while there was significant enhancement to H1975 cells with EGFR L858R + T790M resistance mutations. The elevated MHC-I expression induced by gefitinib or NK cells may block the cytotoxicity of NK cells to A549. Recent report suggests that autophagy caused by chemotherapy can improve tumor cell sensitivity to immunotherapy, which is mediated by up-regulating mannose-6-phosphate receptor on the tumor cell surface [38]. We find that gefitinib can enhance autophagy in the cell lines with L858R + T790M and up-regulate the cell surface MPR expression. MPR antagonist mannose-6-phosphate reduces the cytoxicity of NK cells. The enhanced NK cells cytotoxicity by gefitinib may be attributed to elevated MPR expression induced by gefitinib.

## Conclusions

Our present study suggests that gefitinib has multiple effects on the interaction between NK cells and tumor cells. Similar to imatinib, gefitinib has its own immunomodulatory property, which can enhance NK cell cytotoxicity. Gefitinib enhances NKG2D-NKG2D ligands interaction between NK cells and human lung cancer cells. Combination of gefitinib with NK cells down-regulates stat3 expression. MPR expression induced by gefitinib facilitates antitumor NK cell immunity. Therapeutic significance of our finding is that administration of gefitinib may offer a novel adjuvant strategy to enhance NK cells based immunotherapy in NSCLC with EGFR L858R + T790M resistance mutation.

## Abbreviations

EGFR: Epithelial growth factor; NK cell: Natural killer cell; TKI: Tyrosine kinase inhibitors; MPR: Mannose-6-phosphate receptor; NSCLC: Non-small cell lung cancers.

## Competing interests

The authors declare that they have no competing interests.

## Authors’ contributions

Conception and design: W-YS, H-SS, YT. Development of methodology: W-YS, H-SS, YT, WY. Acquisition of data: H-SS, YT, LD, G-FC, M-XL, Y-TH, S-JH, L-ZQ. Analysis and interpretation of data: W-YS, H-SS, YT, GX, WY. Writing, review, and/or revision of the manuscript: W-YS, H-SS, YT,GX. Administrative, technical, or material support: G-FC, M-XL, Y-TH, S-JH, L-ZQ Study supervision: W-YS, H-SS, YT. All authors read and approved the final manuscript.

## Authors’ information

Sisi He and TaoYin are the co-first authors for this paper.
